# Whole blood as an alternative to peripheral blood mononuclear cell for detection of total HIV-1 DNA

**DOI:** 10.1186/s12879-020-05675-3

**Published:** 2020-12-10

**Authors:** Ling Lin, Yong-Song Yue, Ni-Dan Wang, Lei-Yan Wei, Yang Han, Xiao-Jing Song, Zhi-Feng Qiu, Wei Cao, Tai-Sheng Li

**Affiliations:** 1 Department of Infectious Diseases, Peking Union Medical College Hospital, Peking Union Medical College, Chinese Academy of Medical Sciences, 1# Shuai Fu Yuan, Beijing, 100730 China; 2 Center for AIDS Research, Chinese Academy of Medical Sciences and Peking Union Medical College, Beijing, China; 3 Clinical Immunology Center, Chinese Academy of Medical Sciences, Beijing, China; 4 Tsinghua-Peking Center for Life Sciences, School of Medicine, Tsinghua University, Beijing, China

**Keywords:** AIDS, HIV-1 DNA, Peripheral blood mononuclear cell, Reservoir, Whole blood

## Abstract

**Background:**

A more time saving, convenient, reproducible, and scalable method is needed to assess total HIV-1 DNA levels.

**Methods:**

Frozen whole blood and peripheral blood mononuclear cell (PBMC) samples both 200 μl at the same point were used to detect total HIV-1 DNA. Automatic extraction of total HIV-1 DNA was used to ensure the consistency of sample extraction efficiency. The detection reagent was HIV-1 DNA quantitative detection kit and real-time quantitative PCR was utilized.

**Results:**

Of the 44 included patients, 42 were male and 2 were female, with a median age of 33 years. Thirty-three cases were collected after receiving antiviral treatment, with a median duration of treatment of 3 months, and the other 11 cases were collected before antiviral treatment. The median viral load was 1.83 log10 copies/mL, the median CD4 and CD8 count were 94 and 680 cells/μL, and the median CD4/CD8 ratio was 0.18. The results of the two samples were 3.02 ± 0.39 log10 copies/10^6^ PBMCs in PBMC samples and 3.05 ± 0.40 log10 copies/10^6^ PBMCs in whole blood samples. The detection results of the two methods were highly correlated and consistent by using paired t test (*P* = 0.370), pearson correlation (r = 0.887, *P* < 0.0001) and intra-group correlation coefficient (ICC = 0.887, *P* < 0.0001) and bland-altman [4.55% points were outside the 95% limits of agreement (− 0.340 ~ 0.390)].

**Conclusions:**

The results of the whole blood sample test for total HIV-1 DNA are consistent with those of PBMC samples. In a clinical setting it is recommended to use whole blood samples directly for the evaluation of the HIV reservoir.

## Background

Combined antiretroviral therapy (cART) can effectively inhibit Human immunodeficiency virus-1 (HIV-1) RNA in peripheral blood of infected patients to a low (< 50 copies/mL) or even undetectable levels, but patients need to take the drug for a lifetime, and the virus will rebound immediately upon withdrawal. The reason is that when HIV-1 enters the body, RNA is reverted to DNA and integrated into the genome of infected cells for subsequent life cycle activities. Most of the infected cells die and are eliminated by the immune system after antiviral treatment, while only a small number of infected cells survive and remain at rest, becoming HIV-1 latent reservoir, otherwise known as HIV-1 DNA reservoir [1, 2]. HIV-1 DNA with a complete viral genome can be re-transcribed and translated, resulting in viral rebound after treatment interruption. HIV-1 reservoir is the principal obstacle to cure HIV. Eliminating HIV-1 reservoir has become a heated issue in the field of global HIV research. To achieve this, the first step is to identify a biomarker that can assess the level of the HIV reservoir. In the narrow sense, HIV-1 reservoir refers to a resting CD4 cell in a dormant state but integrates the complete HIV-1 genome and has viral replication capacity [3]. In earlier studies of the HIV-1 reservoir, scientists paid more attention to the concept of narrow HIV-1 reservoir, and the number of CD4 cells that integrated the entire HIV-1 virus and remained at rest.

The total HIV-1 DNA in the cell represents all integrated and non-integrated, genetically complete and defective viruses. Although the detection method of total HIV-1 DNA cannot distinguish defective viruses, more and more studies have shown that defective viruses can also produce viral molecules and then participate in the activation of host cells and the pathogenesis of HIV [4]. There was a good correlation between the total HIV-1 DNA in resting CD4+ T cells and HIV-1 DNA with complete replication capacity [5, 6]. Total HIV-1 DNA can be used as a common biological indicator for assessing the size of HIV-1 reservoir. At present, quantitative polymerase chain reaction (PCR) is mainly used to detect HIV-1 DNA in peripheral blood mononuclear cell (PBMC) or CD4 cells. During the follow-up of HIV/AIDS patients, HIV-1 RNA and CD4 cells were mainly detected as indicators to evaluate the efficacy of cART, while HIV-1 DNA was not a routine test. Researchers often use frozen samples when testing patients’ HIV-1 DNA. PBMC and CD4 cells need to be separated or sorted from fresh whole blood in advance and stored in liquid nitrogen under strict storage conditions, and cell loss will inevitably occur when they are resuscitated.

Avettand-Fènoël et al. detected the HIV-1 DNA level of PBMCs samples, and the results were converted to copies/10^6^ CD4+ T cells or to copies/mL whole blood. The results showed that HIV-1 DNA could predict the progression of the disease regardless of the mode of result expression [7]. But they didn’t test for HIV-1 DNA levels in the whole blood. Another study tested HIV-1 DNA levels in whole blood, but they converted the results using normalization formula, which showed that samples with quite similar data regarding HIV-1 DNA copies/mg DNA had a different HIV-1 DNA/mL of blood [8]. Moreover, the results of this study were not compared with those of PBMCs. In our study, whole blood and PBMCs samples of patients were simultaneously tested at the same time point, and the test results of whole blood samples were converted into units based on the percentage of lymphocytes and monocytes specific in each patient. The results were compared with the total HIV-1 DNA in PBMC in the hope of providing a more time saving, convenient, reproducible, and scalable method for evaluating total HIV-1 DNA levels.

## Methods

The subjects of this study were all HIV-1 infected patients who were followed up in the outpatient department of immunodeficiency in Peking Union Medical College Hospital. Peripheral blood samples were collected during follow-up, and PBMC were separated. Whole blood samples were stored in the refrigerator at − 80 °C, and the PBMC samples were frozen in liquid nitrogen.

### PBMC separation

Fresh peripheral blood was collected with EDTA anticoagulant tube and centrifuged at room temperature. The supernatant plasma was retained then supplemented with normal saline, and the PBMCs were centrifuged out with a lymphocyte separation tubes containing a filter membrane and lymphocyte separation fluid.

### DNA extraction in whole blood and PBMCs

To ensure the consistency of sample extraction efficiency, Roche MagNa Pure nucleic acid extraction instrument was used to extract total HIV-1 DNA automatically with Qiagen QIAsymphony DNA Mini Kits (QIAGEN, Valencia, CA). The sample sizes of whole blood and PBMC were both 200 μL.

### Quantitative detection of HIV-1 DNA

Total cellular human immunodeficiency virus type 1 (HIV-1) DNA in whole blood and PBMCs were amplified and quantified for the HIV-1 long terminal repeat (LTR) gene using a flurescence-based, real-time SUPBIO HIV Quantitative Detection Kit (PCR-fluorescent probe method, Cat. No. Supi-1101, stored at − 20 °C, SUPBIO®, Guangzhou, China). HIV-1 DNA was quantitatively detected by PCR amplification method using HIV-specific primers and HIV-specific probe (Taqman probe) combined with PCR reaction solution, heat-resistant DNA polymerase (Taq enzyme), nucleotide monomer (dNTPs). 7500 real-time quantitative PCR instruments (Applied Biosystems, 7500 Real-Time PCR System) were used. Stage 1: 37 °C × 5 min, 95 °C × 10 min; Stage 2: 95 °C × 15 s, 62 °C × 15 s, 72 °C × 20 s, Cycle 15 times; Stage 3: 95 °C × 15 s, 52 °C × 15 s, 72 °C × 32 s, Cycle 40 times. The quantification range of this assay was 20–5 × 10^6^ copies/10^6^ WBCs.

### Conversion of total HIV-1 DNA test results in whole blood

The results obtained from each quantitative PCR tube included copies of HIV-1 DNA labeled with FAM and copies of human genomic DNA labeled with VIC. The cells in the whole blood are composed of non-nucleated red blood cells and nucleated white blood cells, so the detected human genomic DNA comes from nucleated white blood cells (WBC). Therefore, the total HIV-1 DNA detection result obtained by this quantitative PCR method = (copies of HIV-1 DNA/copies of WBCs) × 10^6^, that is, the measurement unit of HIV-1 DNA is “copies of HIV-1 DNA per million white blood cells (copies/10^6^ WBCs)”. However, “copies of HIV-1 DNA per million peripheral blood mononuclear cells (10^6^ PBMCs)” is commonly used in the international currently. To maintain consistency, we converted the test results. Universal result = original result/(lymphocytes% + monocytes%).

### Statistics

All measurement data were analysed for normality. Mean ± standard deviation (SD) was used for data in accordance with normality distribution, while median ± interquartile range (IQR) was used for data inconsistent with normality distribution. In this study, paired t test, pearson correlation analysis, intra-group correlation coefficient, and bland-altman were used to compare the results of total HIV-1 DNA from two-types samples harvested at the same time, the statistical methods used included. Statistical analysis was performed using SPSS 19.0 (IBM Corporation, Armonk, New York, USA), GraphPad Prism 8.0 (GraphPad Software, Inc. La Jolla, CA, USA) and Medcalc 19.4. A *p* value < 0.05 was considered statistically significant.

## Results

### Basic characteristics at enrollment

**Fig. 1:**
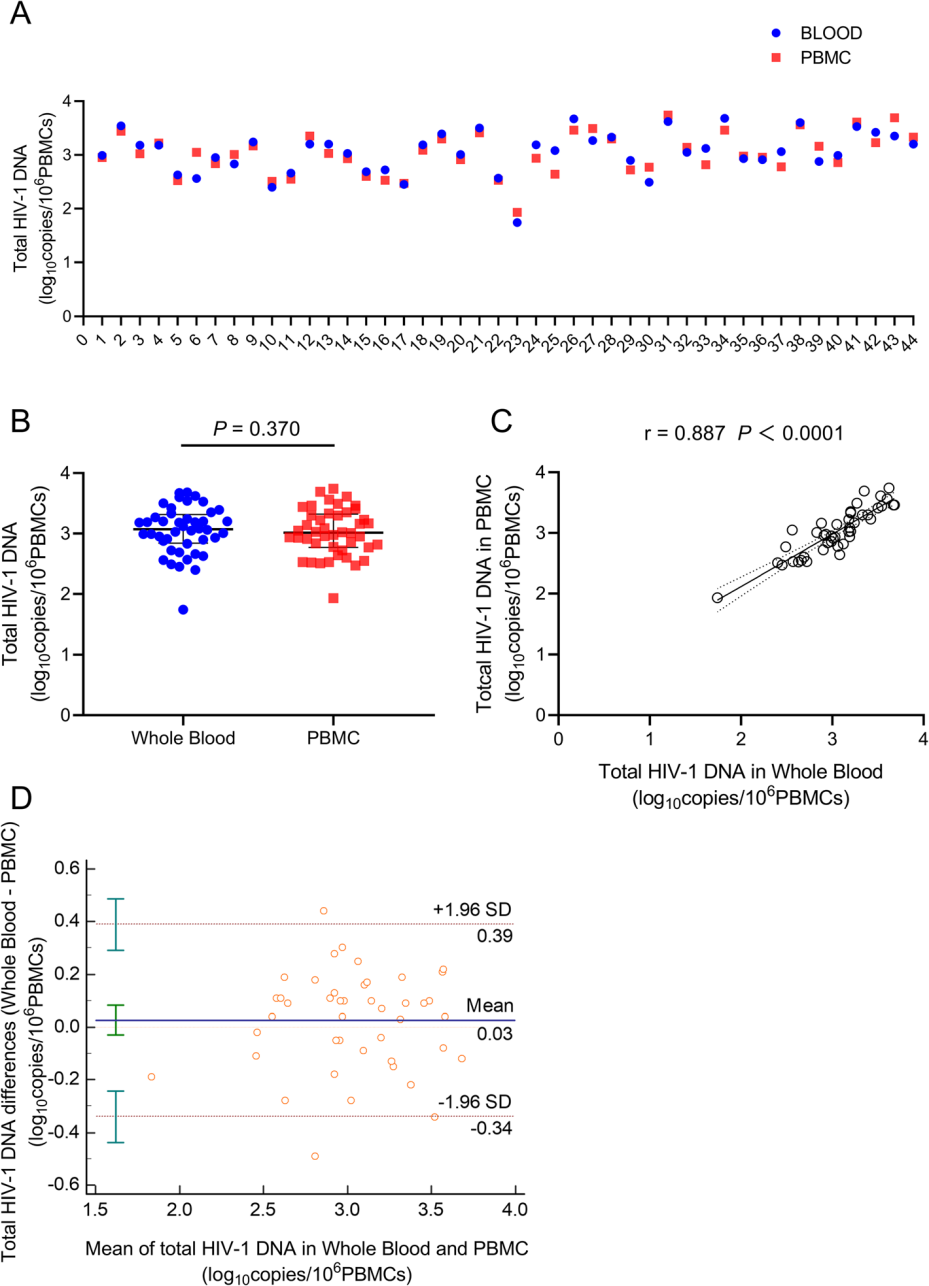
Comparison of total HIV-1 DNA test results between PBMC and whole blood Samples (*N* = 44). **a** Detection values of total HIV-1 DNA in PBMC and whole blood samples from 44 cases. **b** Mean comparison of total HIV-1 DNA between PBMC and whole blood group. The statistical method was Paired T test. **c** Correlation between total HIV-1 DNA test results in PBMC and whole blood group. The statistical method was Pearson correlation analysis. **d** Consistency between PBMC and whole blood total HIV-1 DNA test results, statistical method was bland-altman. The horizontal coordinate is the mean value of HIV-1 DNA test results between whole blood and PBMCs, and the vertical coordinate is the difference value of HIV-1 DNA test results between whole blood and PBMCs. The solid blue line in the figure is the mean value of the difference between the detection results of the two samples (Mean = 0.04). The top and bottom dotted lines are the 95% limits of agreement (95% LoA). PBMC: peripheral blood mononuclear cell

This study is a retrospective study, a total of 44 outpatients of chronic HIV-1 infected, including 42 males and 2 females, with a median age of 33 years were included. Of these, 33 cases were collected after antiviral treatment, the median treatment time was 3 months, and 11 cases were collected before antiviral treatment. The median viral load was 1.83 log10 copies/mL, the median CD4 count was 94 cells/μL, the median CD8 count was 680 cells/μL, and the median CD4/CD8 ratio was 0.18 (Table 1). Forty-four samples of whole blood and PBMCs (both frozen samples) collected at the same time point were extracted and tested for total HIV-1 DNA. The results of total HIV-1 DNA detected with PBMCs and whole blood were very similar (Fig. 1a).

**Table 1:** The patient characteristics of the tested samples

Characteristics	Value
Gender (%)	
Male	42 (95.5%)
Female	2 (4.5%)
Age (years, IQR)	33 (27, 40)
cART time (months, IQR)	
11 cases before cART	0
36 cases after cART	3 (2, 8)
HIV-1 RNA (log10copies/mL, IQR)	1.83 (1.30, 4.46)
CD4 counts (cells/μL, IQR)	94 (70, 369)
CD8 counts (cells/μL, IQR)	680 (501, 826)
CD4/CD8 ratio (IQR)	0.18 (0.09, 0.34)

*IQR* Interquartile range

### Total HIV-1 DNA in whole blood and PBMC samples were highly correlated and were not different between the mean of two groups

Paired t test was used to compare the mean values between the two groups (Fig. 1b). The mean ± SD of total HIV-1 DNA detected by PBMCs was 3.02 ± 0.39 log10 copies/10^6^ PBMCs, and the mean ± SD of total HIV-1 DNA detected by whole blood was 3.05 ± 0.40 log10 copies /10^6^ PBMCs. The *P* value of the paired t test was 0.370, indicating no significant statistical difference between these two groups.

We further compared the correlation of total HIV-1 DNA results between the two-types samples for the individual. A pearson correlation analysis showed that the correlation coefficient (r) of the total HIV-1 DNA test results of two-types samples was 0.887, *P* < 0.0001 (Fig. 1c).

### Total HIV-1 DNA in whole blood and PBMC samples were consistent

The third statistical method is intra-class correlation coefficients (ICC), also known as Kappa test, with a coefficient of 0.887, *P* < 0.001(95% confidence interval is 0.803–0.937). The coefficient range of ICC is 0–1. The larger the ICC value is, the smaller the systematic error and random error of the two groups of measurement differences, and the data consistency is good. It generally considers that consistency is better when ICC value ≥0.75. Therefore, this method shows that the detection results of whole blood and PBMC samples are consistent.

In theory, two-types samples generally do not get exactly the same results, but there will be a trend different. This kind of systematic error is also known as “bias”. The magnitude of the “bias” can be estimated by the mean (d) of the difference measured by the two-types samples, and the variation of mean (d) can be described by the SD of the difference. When the difference between the measured results is normality distribution, the 95% margin should be between mean (d) ± 1.96 SD, which is called the 95% limits of agreement (95% LoA). Since the difference between the measurement results of the two methods is related to the measurement results of the two methods, the abscissa (X-axis) is the average value of the measurement results of the two methods, and the ordinate (Y-axis) is the difference of the measurement results of the two methods. The mean difference between the test results of the two-types samples was 0.030 log10 copies/10^6^ PBMCs. As can be seen from bland-altman diagram, 4.55% of the points (2/44) are beyond 95% LoA (−0.340 ~ 0.390).

## Discussion

HIV-1 DNA is exist in the peripheral blood of a variety of cells that can be infected with HIV-1, including monocytes and all CD4 cell subsets. Cells with a high level of HIV-1 DNA content in chronic HIV-infected patients are central memory CD4 cells and transitional state memory CD4 cells [9]. This type of cell has a strong ability to proliferate and a long half-life, which make HIV-1 DNA stable for a long time.

However, in the early stage of infection, a large number of T cells are activated to induce effector memory T cells to play an immune role. Effector memory CD4 cells become one of the main cells in the distribution of HIV-1 DNA [10]. In fact, in animal models and humans, the largest reservoir is in lymph nodes and associated intestinal lymphoid tissue [11]. In the acute phase and early stage of infection, the content of HIV-1 DNA in CD4 cells of intestinal lymphoid tissue was 10 times higher than that in peripheral blood [3]. A study on human intestinal lymphoid tissue and HIV-1 DNA in whole blood showed a good correlation between them [12]. In addition, astrocytes in the central nervous system, epithelial cells of the reproductive tract, and other organs or body fluids also contain HIV-1 DNA [13–16].

Previous studies have shown that LTR has been proved to be the best target for DNA quantification [17]. In this study, real-time fluorescence based HIV detection kit was used to amplify and quantify HIV-1 DNA using LTR gene primers, which ensured the reliability and accuracy of HIV-1 DNA detection. The high genetic diversity of HIV-1 represented by different subtypes may lead to the inaccuracy of HIV-1 DNA quantification [18]. In the large number of HIV infected samples we tested previously [19, 20], the method was used to detect HIV-1 DNA in patients independent of subtypes. The quantification range for HIV-1 DNA using this assay was 20 to 5 × 10^6^ copies/10^6^ WBCs in our study. It has a wide range that is not worse than using by COBAS®AmpliPrep [21]. Although HIV infection includes HIV-1 and HIV-2, most patients are infected with HIV-1, and our research is focused on HIV-1 infected patients. This may be a limitation of our study, but it will not have a significant impact on the results of the study.

We used whole blood samples and PBMCs samples of 44 patients to detect total HIV-1 DNA, and observed that the total HIV-1 DNA test results of the two-types samples were very close. We used four different statistical methods to test the consistency of the two samples. The results of paired t test showed that there was no statistically significant difference between the two groups in total HIV-1 DNA test results. However, the paired t test mainly compares the difference in mean value, which can only reflect that the overall mean value may be the same. The essence of paired t test is “difference” test, not “consistency” test, so the results of the paired t test cannot reflect the consistency of the data. The Pearson correlation coefficient results showed that the correlation coefficient r value of the two-types sample test results was 0.887, indicating that the two-types sample test results were highly correlated. The pearson correlation coefficient is now widely used to evaluate the consistency, but it reflects the closeness of the linear relationship between the two variables, and is not strictly consistent. The ICC and bland-altman method are the real statistical methods that can reflect the consistency of the two results. Our results showed that the ICC of the total HIV-1 DNA test results of two-types samples was 0.887, and the data consistency was good. Bland-altman method showed that the 95% LoA of the two groups was − 0.340 to 0.390. It means that as long as the difference between the results of two groups is within 0.390 log^10^ copies/10^6^PBMCs, the detection results can be considered to be consistent. 3 = 0.48 log^10^, 2 = 0.30 log^10^, which means that the difference of HIV-1 DNA detected by two different types of samples is 2–3 copies/10^6^ PBMCs. In other words, the consistency of the test results of the two groups was good.

## Conclusions

In this study, we used 200 μL whole blood to detect total HIV-1 DNA directly thawed from a − 80 °C refrigerator. However, the separation of PBMCs requires more peripheral blood, and the isolation and preservation procedures for PBMCs are complex, time-consuming and expensive. Our study measured total HIV-1 DNA levels in whole blood and PBMCs samples from patients’ two-types samples at the same follow-up point and found a high degree of consistency. The HIV-1 DNA results from the whole blood sample were converted into international common units based on the percentage of lymphocytes and monocytes specific in each patient, which not only made the results better reflect the real HIV-1 DNA level in individual’s but also comparable to the results detected by PBMCs. Therefore, whole blood testing for total HIV-1 DNA is more convenient, economic and operational, and can replace PBMCs samples. In conclusion, the results of whole blood and PBMCs samples showed no difference, good correlation and consistency. For the quantitative study of total HIV-1 DNA with a large sample size, it is a better choice to extract DNA from whole blood and test HIV-1 DNA.

## Data Availability

Datasets used in this analysis are available from the corresponding author upon request.

## Abbreviations

cART: Combined antiretroviral therapy
HIV-1: Human immunodeficiency virus-1
PCR: Polymerase chain reaction
PBMC: Peripheral blood mononuclear cell
WBC: White blood cells
SD: Standard deviation
IQR: Interquartile range
ICC: Intra-class correlation coefficients
LoA: Limits of agreement
